# Calming the (Cytokine) Storm: Dimethyl Fumarate as a Therapeutic Candidate for COVID-19

**DOI:** 10.3390/ph14010015

**Published:** 2020-12-26

**Authors:** Cara A. Timpani, Emma Rybalka

**Affiliations:** 1 Institute for Health and Sport, Victoria University, Melbourne, VIC 8001, Australia; emma.rybalka@vu.edu.au; 2 Australian Institute for Musculoskeletal Science, St Albans, VIC 3021, Australia

**Keywords:** COVID-19, dimethyl fumarate, Nrf2, therapeutics

## Abstract

COVID-19 has rapidly spread worldwide and incidences of hospitalisation from respiratory distress are significant. While a vaccine is in the pipeline, there is urgency for therapeutic options to address the immune dysregulation, hyperinflammation and oxidative stress that can lead to death. Given the shared pathogenesis of severe cases of COVID-19 with aspects of multiple sclerosis and psoriasis, we propose dimethyl fumarate as a viable treatment option. Currently approved for multiple sclerosis and psoriasis, dimethyl fumarate is an immunomodulatory, anti-inflammatory and anti-oxidative drug that could be rapidly implemented into the clinic to calm the cytokine storm which drives severe COVID-19.

## 1. Introduction 

The severe acute respiratory syndrome coronavirus type-2 (SARS-CoV-2) is responsible for the COVID-19 pandemic. Transmission, infection and mortality rates are significant (particularly compared to seasonal influenza) indicating the immediate need for a vaccine. Since it is currently unclear when an effective vaccine will be widely available and there are no standard-of-care treatment options for COVID-19, there is high unmet clinical need for therapeutics that can rapidly translate to improve patient care and reduce mortality rates. 

**Figure 1:**
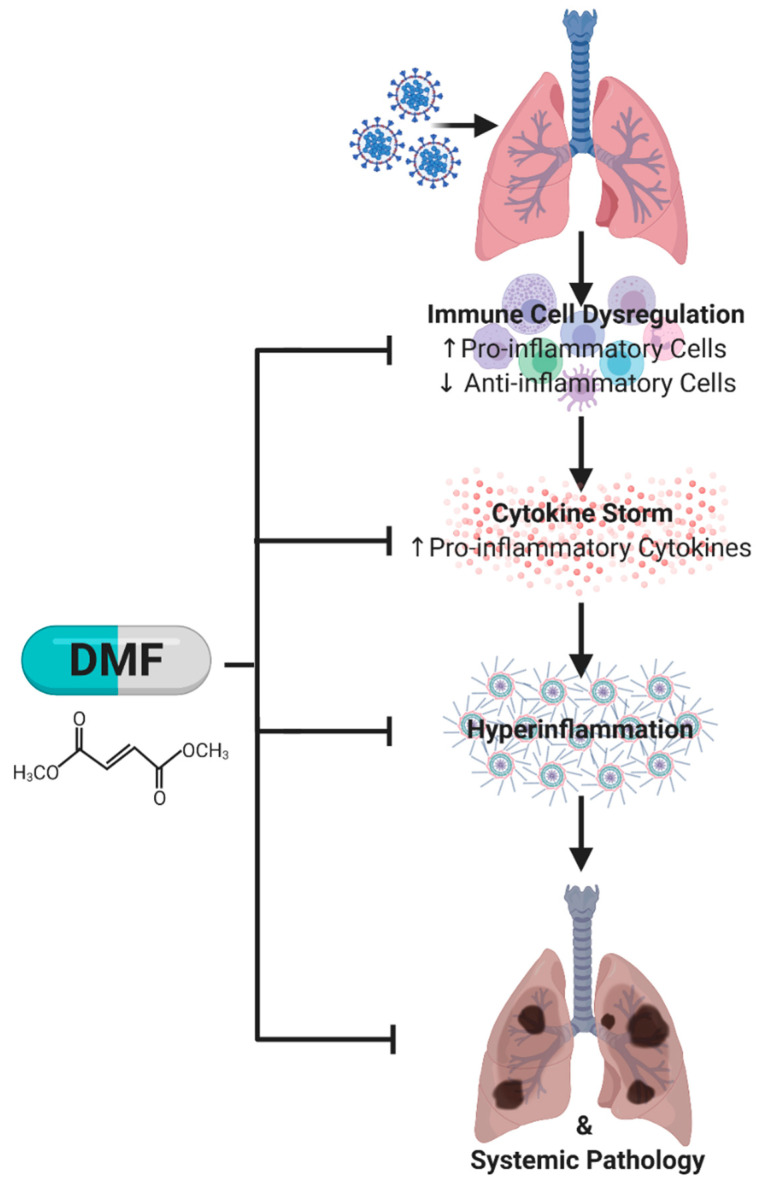
Simplified pathological pathway to lung (and systemic) injury in severe cases of COVID-19. Infection with the SARS-CoV-2 virus can lead to a dysregulated immune response in which pro-inflammatory cells dominate the immune cell population. These pro-inflammatory cells intensify cytokine production and release resulting in hyperinflammation. This hyperinflammatory state promotes lung (and systemic) pathology, which correlates with poorer prognosis. It is well documented that dimethyl fumarate (DMF) can modulate immune cell populations to shift the ratio of anti-inflammatory to pro-inflammatory cytokine production and release, which in turn reduces hyperinflammation and subsequent tissue injury.

Clinical presentation of COVID-19 varies but typical symptomology includes fever, cough and fatigue. In most cases hospitalisation is not required; however, for the elderly and those with co-morbidities (i.e., diabetes, cardiovascular disease, obesity, respiratory disease), the likelihood of hospitalisation (and mortality) increases significantly [1,2]. Severe cases usually present with acute respiratory distress syndrome (ARDS), the result of dysregulated host immune response to the virus [3]. The outcome of this immuno-dysregulation, which may be driven by underlying inflammation associated with age and co-morbidities, is a cytokine storm, i.e., elevated interferon (IFN), interleukins (IL), tumour necrosis factor-α (TNF-α), amongst others. The cytokine storm potentiates hyperinflammation, oxidative stress and haematological changes including lymphopenia, thrombocytopenia and macrophage activation syndrome. Collectively, these pathologies exacerbate the dysregulated host response and cause significant tissue injury to lung (and other) tissues resulting in respiratory (and often multi-organ) failure (Figure 1) [4]. Given the widespread organ/system assault in severe cases of COVID-19, there is clinical need for a therapeutic which addresses the multifactorial pathogenesis to induce systemic cytoprotection and re-establish host responsivity. Dimethyl fumarate (DMF), an approved drug with immunomodulatory, anti-oxidative and anti-inflammatory properties in all tissues, is one potential treatment that could be rapidly implemented into the clinic. Here, we provide perspectives on the potential re-purposement of DMF to treat the cytokine storm caused by severe COVID-19. We have searched the National Library of Medicine Pubmed^®^ database from 20 August 2020 to 14 November 2020 using the key search terms “dimethyl fumarate”, “severe inflammation”, “cytokines”, “immunomodulation” and “COVID-19/SARS-CoV-2” and included in our discussion, those papers which match the mode of action of DMF with the symptomology of the cytokine storm induce by SARS-CoV-2.

## 2. Main

**Figure 2:**
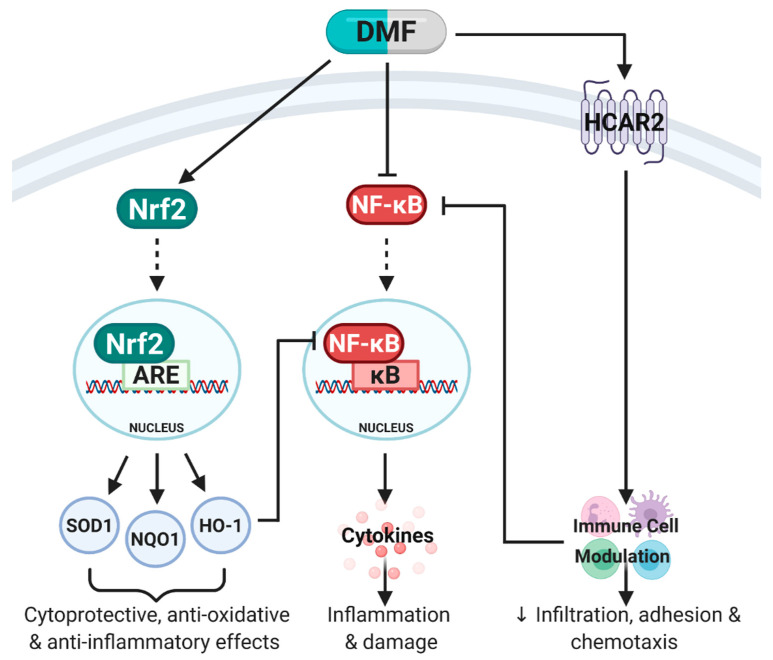
Simplified schematic of pathways activated by dimethyl fumarate (DMF). DMF is known to mediate its anti-inflammatory, anti-oxidative and immunomodulatory effects primarily through three molecular pathways: (1) Activation of nuclear factor erythroid 2-related factor 2 (Nrf2) which binds to the antioxidant response element (ARE) in the nucleus to stimulate transcription of Phase II enzymes including superoxide dismutase (SOD1), NAD(P)H quinone oxidoreductase-1 (NQO1) and heme oxygenase-1 (HO-1). Together, SOD1, NQO1 and HO-1 mediate cytoprotective, anti-oxidative and anti-inflammatory effects. (2) DMF can directly inhibit nuclear factor kappa B (NF-κB), which prevents the translocation of NF-κB into the nucleus, binding to the κB site, release of pro-inflammatory cytokines and subsequent inflammation and damage. DMF can also indirectly inhibit NF-κB through HO-1 expression and immune cell modulation through hydroxycarboxylic acid receptor 2 (HCAR2) activation. (3) DMF’s immunomodulatory effects are predominantly mediated through HCAR2 activation which modulates immune cell populations (pro-inflammatory to anti-inflammatory shift) and inhibits NF-κB. Adapted from [7]. Created with BioRender.com.

DMF is a methyl ester of fumaric acid (chemical formula C_6_H_8_O_4_) that is hydrolysed in the small intestine to the active metabolite monomethyl fumarate [5,6,7,8]. DMF is a potent activator of the nuclear factor erythroid 2-related factor 2 (Nrf2) pathway which modulates inflammation and oxidative stress by upregulating cellular defence mechanisms (i.e., cytoprotection through Phase II antioxidant expression, chiefly superoxide dismutase (SOD1), NAD(P)H quinone oxidoreductase-1 (NQO1) and heme oxygenase-1 (HO-1; Figure 2) [6,7,8,9]. In addition, DMF exerts some of its effects through Nrf2-independent mechanisms: (1) indirect inhibition of the inflammatory mediator, nuclear factor kappa B (NF-κB); and (2) hydroxycarboxylic acid receptor 2 (HCAR2) activation, which modulates immune cell (particularly neutrophil) infiltration, adhesion and chemotaxis, reduces pro-inflammatory cytokine production and inhibits NF-κB (Figure 2) [6,8,9,10,11,12]. DMF is clinically indicated for Multiple Sclerosis (MS) and psoriasis, diseases characterised by immune dysregulation, inflammation and oxidative stress [6]. Broadly, DMF treatment of these diseases: (1) Drives anti-inflammatory immune cell composition (Table 1; for a detailed review see [13]); (2) increases the ratio of anti- to pro-inflammatory cytokines; (3) induces the anti-oxidative and cytoprotective response through Nrf2; and (4) inhibits NF-κB to convert T helper (Th) cells from the Th1/17 subset to the anti-inflammatory Th2 subset [14,15,16,17,18,19,20,21,22,23,24,25,26,27,28,29,30,31]. Collectively, these biological activities mitigate the severe pro-inflammatory and -oxidative mechanisms in both MS and psoriasis, which reduces disease progression to impart significant clinical impact.

The SARS-CoV-2 virus has been demonstrated to affect a significant number of immune cell populations including T cells, B cells, natural killer cells, monocytes, eosinophils and basophils [38,39,40,41,42]. These populations typically decrease in number (the severity of the disease dictates the magnitude of reduction), albeit pro-inflammatory phenotypes dominate the remaining immune cell population. Characteristic of the pro-inflammatory immune response is the increase in neutrophil-to-lymphocyte ratio (NLR). Neutrophilia and lymphopenia, which increase the NLR, are associated with severe viral infection and correlate with a poorer prognosis [43,44,45,46,47]. Moreover, in severe cases, the elevated neutrophil count is correlated with the formation of neutrophil extracellular traps (NETs). NETs are an important innate immunity defense mechanism as they trap and kill pathogens; however, their dysregulation induces oxidative stress (through reactive oxygen species (ROS) production), inflammation, damage, thrombosis and fibrosis to the surrounding tissues. It has been documented that neutrophils infiltrate the lungs [48,49,50] and induce elevated NET formation [48,49,50,51,52,53,54] in severe cases of COVID-19. DMF has been shown to modulate neutrophil counts [12,55] and NET formation [56,57]. Importantly, DMF reduces neutrophil adhesion, migration and infiltration [12,55,56,58] and neutrophil-induced ROS production [56] indicating that DMF can moderate the pro-inflammatory (and oxidative) effects of a dysregulated neutrophil response. In addition to DMF’s modulatory effects on various immune cell populations to shift from pro-inflammation to anti-inflammation, DMF also modifies an extensive cytokine profile [16,20,22,23,59], which is consistent with that observed in the cytokine storm characteristic of COVID-19 (i.e., granulocyte-colony stimulating factor (CSF), granulocyte-macrophage-CSF, IFN-γ, interferon-γ-inducible protein-10 (IP-10), IL-1β, IL-6, macrophage inflammatory protein (MIP)-1α, MIP-1β, monocyte chemoattractant protein-1 (MCP-1), TNF-α; see Table 2) [3,40,46,60,61,62]. Given the cytokine storm is strongly correlated with poorer prognosis [40,61,62], calming it is a logical approach. In this regard, the corticosteroid, dexamethasone, which shares some comparable immunosuppressive properties as DMF, has been used successfully in the clinical treatment of COVID-19 [63,64]. However, where corticosteroids elicit immunosuppression mainly through sequestration of CD4+ T-lymphocytes in the mononuclear phagocyte system and by inhibiting cytokine and lymphokine transcription (especially IL-1 and IL-6) [65], DMF modulates a more extensive cytokine profile as well as potent anti-oxidation activity. Of note, there is evidence that DMF induces lymphopenia in some MS patients [66] and, as such, diligent monitoring would be pertinent to ensure DMF does not exacerbate the lymphopenia documented in severe COVID-19 cases [43]. Despite this, DMF is generally well tolerated and is clinically approved indicating scope for rapid clinical translation. Importantly, the overall anti-inflammatory and anti-oxidative phenotype induced by DMF in MS and psoriasis patients would be beneficial for COVID-19 patients given the similar pathological mechanisms which advance disease severity and progression. 

SARS-CoV-2 uses angiotensin converting enzyme II (ACE2) as its cellular entry receptor [67,68]. The binding of SARS-CoV-2 to ACE2 receptors is likely to reduce ACE2 receptor binding ability and attenuate downstream signalling from anti-inflammatory to pro-inflammatory pathways [69,70,71,72,73,74,75]. In the lungs, pro-inflammatory ACE2 signalling mediates immune cell infiltration, inflammation, injury and fibrosis [70,71,72,73,74,75]. While there is little research into the effect of DMF on ACE2 signalling, there is evidence that DMF can promote anti-inflammatory ACE2 signalling since it reduces inflammatory mediators (NF-κB-derived) and cytokine production in a murine model of acute lung injury [76]. In support of Nrf2 playing a role in ACE2 signalling, pharmacological Nrf2 inhibition decreases ACE2 mRNA [77]. Recent modelling also demonstrates that SARS-CoV-2 interacts with nicotinic acetylcholine receptors which may inhibit the cholinergic anti-inflammatory pathway and mediate COVID-19 pathology [78]. Consistent with this, the incidence of hospitalisation in smokers with COVID-19 is lower than predicted (albeit hospitalised smokers have poorer prognosis [79,80]) indicating that binding of nicotine to the receptor may competitively obstruct SARS-CoV-2 virulence [81]. DMF has been shown to facilitate cholinergic stimulation in MS patients [82] suggesting that, in the presence of SARS-CoV-2, DMF may competitively bind nicotinic acetylcholine receptors to reduce COVID-19 pathogenesis in the first instance. This is consistent with only several reported cases of COVID-19 in DMF-treated MS patients and with none of them having significant symptoms that required hospitalization [83]. 

There is growing evidence that the SARS-CoV-2 virus impedes the homeostatic response to restore redox balance. Nrf2, and its associated downstream antioxidant genes, are significantly reduced in lung biopsies of COVID-19 patients [3,84] suggesting Nrf2 suppression is a mechanism for SARS-CoV-2 replication. However, as with other conditions where Nrf2 induction is suppressed (e.g., aging-related sarcopenia), pharmacological rebalancing of Nrf2 responsiveness to noxious stimuli is possible. *In vitro* treatment of a SARS-CoV-2 infected Calu3 lung cancer cell line with the potent Nrf2 activator, DMF: (1) exerted an anti-viral effect through inhibition of SARS-CoV-2 replication; (2) reduced the pro-inflammatory cytokine profile; and (3) increased heme oxygenase-1 (HMOX1) gene expression [84]. HMOX1 gene upregulation (and HO-1 production) is associated with anti-viral activity against many viruses including influenza [85,86,87,88,89,90,91,92,93,94] and promotes the anti-inflammatory macrophage phenotype and anti-inflammatory IL-10 cytokine production [95,96]. Indeed, DMF is a well-known inducer of HO-1 [92,97,98,99,100,101,102,103,104,105,106]. Furthermore, previous literature indicates protective effects of DMF on respiratory pathologies including: (1) mitigation of the allergic asthma response [107,108]; (2) inhibition of airway smooth muscle cell proliferation (associated with airway remodeling) [109]; (3) reduction of lung inflammation [99,107,108,110,111] and cytokine production [111,112,113] and; (4) prevention of lung fibrosis in pulmonary arterial hypertension [99]. These findings emphasize the importance of the Nrf2 pathway in SARS-CoV-2 virulence and the potential therapeutic capacity of DMF for COVID-19 treatment, as recently suggested by [114]. 

## 3. Conclusions

Marked by immune dysregulation, hyperinflammation and oxidative stress, severe cases of COVID-19 may benefit from the immunomodulatory, anti-inflammatory and anti-oxidative properties of DMF. However, caution must be taken—the immunosuppressive effect of DMF may be counterproductive to mounting the host anti-viral immune response in the early stages of COVID-19 and, therefore, may expediate virulence. Thus, DMF may only be suitable for severe, progressed cases of COVID-19. 

## Figures and Tables

**Table 1:** Overview of the effect of dimethyl fumarate on T and B cells in relapsing–remitting multiple sclerosis patients.

Patient Number	Age Range (years)	Length of DMF Treatment	Additional Medication during DMF Treatment	Effect on T & B Cells	Ref
15 (7F/8M)	24–54 (median 40.7)	6 m	-	*T cells*: ↓ Th1 & Th17 cells, ↑ CD4 and CD8 naïve cells, ↓ CD4 and CD8 memory cells, ↓ CD8 cells	[16]
13 (11F/2M)	20–60 (median 41)	Not stated	-	*B cells*: ↓ B cell number, ↓ memory B cells, ↑ naïve B cells, ↓ pro-inflammatory B cells (GM-CSF+, IL-6+, TNF-α+), ↓ pro-inflammatory co-stimulatory molecules (CD80+)	[17]
20 (16F/4M)	43 ± 8	4–6 m	-	*T cells*: ↑ Th2/Th1Th17 ratio, ↓ memory T cells, ↑ naïve T cells, ↓ CD4 and CD8 cells, ↓ pro-inflammatory T cells (IFN-γ+), ↑ anti-inflammatory T cells (IL-4+) *B cells*: ↓ B cell number	[25]
18 (14F/4M)	43 ± 9	18–26 m	-	*T cells*: ↓ Th1 & Th17 cells, ↑ Th2 cells, ↑Th2/Th1Th17 ratio, ↓ CD4 and CD8 cells, ↓ memory T cells, ↑ naïve T cells, ↓ pro-inflammatory T cells (IFN-γ+, IL-17+), ↑ anti-inflammatory T cells (IL-4+) *B cells*: ↓ B cell number	
18 (13F/5M)	43.9 ± 10.8	6 m	-	*B cells*: ↓ memory B cells, ↑ naïve B cells, ↓ pro-inflammatory B cells (GM-CSF+, IL-6+, TNF-α+)	[22]
24 (21F/3M)	24–63 (median 44.6)	≥6 m	-	*T cells*: ↓ Th1 cells, ↓ CD4 and CD8 memory cells, ↑ CD4 and CD8 naïve cells	[26]
43 (31F/12M)	46±11	15 ± 9 m	-	*T cells*: ↓ CD8 memory cells, ↑ CD8 naïve cells, ↓ pro-inflammatory T cells (GM-CSF+, IFN-γ+, TNF-α+), *B cells*: ↓ pro-inflammatory co-stimulatory molecules (CD80+)	[27]
13 (8F/5M)	Female: 31–58 (median 46.5) Male:33–57 (median 35)	4–6 m	One patient tapered off steroids for first 6 weeks of DMF treatment	*B cells*: ↓ B cell number, ↓ memory B cells	[30]
13 (11F/2M)	20–60 (median 41)	0–12 m	-	*T cells*: ↓ CD4 and CD8 cells, ↓ memory T cells, ↑ naïve T cells, ↓ pro-inflammatory T cells (IFN-γ+)	[32]
20 (11F/9M)	26–60 (median 41)	0–12 m	-	*T cells*: ↓ T cell number, ↓ CD4 and CD8 cells, ↑ anti-inflammatory Treg cells, ↓ memory T cells, ↑ naïve T cells	[33]
25 (48% F/52% M)	35.4 ± 11.1	At least 3 m	-	*T cells*: ↓ CD8 cells *B cells*: ↓ memory B cells, ↓ pro-inflammatory B cells (IL-6+, TNF-α+), ↓ pro-inflammatory co-stimulatory molecules (CD 40+, CD69+, CD80+, CD86+)	[34]
35 (71.4% F/28.6% M)	21–67 (mean 46.1)	0–12 m	-	*T cells*: ↓ T cell number, ↓ CD4 and CD8 cells *B cells*: ↓ B cell number	[35]
51 (35F/16M)	34.8 ± 10.8	6 m	Methylprednisone-treated patient samples collected 4 weeks after last administration	*T cells*: ↓ T cell number, ↓ CD4 and CD8 cells *B cells*: ↓ B cell number	[29]
43 (28F/15M)	38 ± 2	15 w	-	*T cells*: ↑ transitional T cells *B cells*: ↓ memory B cells, ↑ naïve B cells, ↑ anti-inflammatory B cells (IL-4+, IL-10+, TGF-β+), ↓ pro-inflammatory co-stimulatory molecules (CD69+, CD80+, CD86+)	[36]
21	25–50 (median 37)	12 m	-	*T cells*: ↓ T cell number, ↑ transitional T cells *B cells*: ↓ B cell number, ↓ memory B cells, ↑ naïve B cells,	[37]

Abbreviations: DMF: Dimethyl fumarate; GM-CSF: Granulocyte-macrophage-colony stimulating factor; IFN-γ: Interferon-γ; IL: Interleukin; Th: T helper cells; Treg: T regulatory cells; TGF-β: Transforming growth factor-β; TNF-α: Tumour necrosis factor-α.

**Table 2:** Overview of the effect of dimethyl fumarate on cytokine production.

Elevated Cytokines in COVID-19 Patients	Effect of DMF	Model/Disease
G-CSF [40,61,62]	↓	Murine splenocytes [59], Human primary ASMCs [110]
GM-CSF [40,61,62]	↓	Human RRMS PBMCs [16,17,18,22], Human Psoriatic PBMCs [24], Murine splenocytes [59,115], Human UVECs [116], Murine BMDMs [117], Murine EAE [118]
Gro-1α [62]	↓	Human keratinocytes & PBMCs [23], Murine hepatic injury & Kupffer cells [119], Human UVECs [120]
IFN-γ [40,61,62]	↓	Murine EAE [8,104,118], Human RRMS PBMCs [16,18,19,21,25], Human psoriatic keratinocytes [20], Human psoriatic PBMCs [24], Murine ischaemic stroke model [93], Murine EAN & macrophage cell line [94], Human psoriatic T cells [104], Murine splenocytes [115,121], Murine BMDCs & allogeneic splenic T cell co-culture [122], Human PBMCs [123], Murine BMDCs [124]
IL-1α [61,62]	↓	Murine splenocytes [115], Primary human keratinocytes & PBMCs [125]
IL-1β [40,61,62]	↓	Murine splenocytes [59], Murine ischaemic stroke model [93], Murine hepatic injury & Kupffer cells [119], Primary human keratinocytes & PBMCs [125], Murine colitis model [126], Murine epilepsy model [127], Primary murine microglial & astroglial co-cultures [128,129], Murine SCD model [130], Murine intracerebral hemorrhage models [131]
IL-2 [40,61,62]	↓	Murine EAE [8], Murine splenocytes [121], Murine BMDCs & allogeneic splenic T cell co-culture [122], Human PBMCs [123], Primary human & murine T cells [132]
IL-4 [40,61]	↑	Murine EAE [8,104], Human PBMCs [24], Human RRMS PBMCs [25], Murine EAN & macrophage cell line [94], Human psoriatic T cells [104], Murine spinal cord damage model [133]
IL-6 [3,40,61,62]	↓	Human RRMS PBMCs [17,21,22], Human psoriatic keratinocytes [20], Murine splenocytes [59,115,121], Murine EAN & macrophage cell line [94], Primary human asthmatic ASMCs [111], Human UVECs [116,120], Murine BMDMs [117], Murine BMDCs & allogeneic splenic T cell co-culture [122], Human PBMCs [123], Murine BMDCs [124], Primary human keratinocytes & PBMCs [125], Murine colitis model [126], Murine epilepsy model [127], Primary murine microglial & astroglial co-cultures [128,129], Primary human & murine astrocyte cultures [134], Murine & primate SCD models [130,135], Murine renal/liver I/R injury model [136,137], Primary human lung fibroblasts [138], Murine experimental sepsis [139], Murine IDD model [140]
IL-8 [3,40,61]	↓	Human keratinocytes & PBMCs [23], Murine osteoblastic cells [106], Human UVECs [120], Murine IDD model [140], Human mast cell line & primary CBDMCs [141]
IL-10 [40,61,62]	↑	Human RRMS PBMCs [30], Murine EAN & macrophage cell line [94], Human psoriatic T cells & murine EAE [104], Murine intracerebral hemorrhage models [131]
IL-12p40 [61,62]	↓	Human psoriatic T cells & murine EAE [104], Human PBMCs [123], Murine BMDCs [124], Murine primary microglia [142]
IL-12p70 [62]	↓	Murine ischaemic stroke model [93], Human PBMCs [123,143]
IL-13 [40,61,62]	↓	Murine BMDMs [117], Murine splenocytes [115]
IL-17 [40]	↓	Murine EAE [8,104], Human PBMCs [24], Human RRMS PBMCs [18,25,144], Murine ischaemic stroke model [93], Murine EAN & macrophage cell line [94], Human psoriatic T cells [104], Murine splenocytes [121], Murine BMDCs [124]
IL-18 [61,62]	↓	Murine SCD model [130], Murine renal I/R injury model [136]
IP-10 [40,62]	↓	Human keratinocytes & PBMCs [23], Murine splenocytes [59], Murine ischaemic stroke model [93], Human primary ASMCs [110,112], Primary human & murine astrocyte cultures [134], Human tumour biopsies, cancer cell lines & oncolytic viruses [145]
MCP-1 [3,40,61]	↓	Murine splenocytes [59], Human HIV-infected monocyte-derived macrophages [92], Human UVECs [116,120], Primary human & murine astrocyte cultures [134], Primary murine microglia [142]
MCP-3 [62]	↓	Murine liver I/R injury model [137]
MIG [3,61,62]	↓	Human keratinocytes & PBMCs [23]
MIP-1α [40,61,62]	↓	Murine splenocytes [59]
MIP-1β [62]	↓	Murine splenocytes [59]
PDGF-BB [40,61,62]	↓	Human UVECs [115]
RANTES [61]	↓	Murine splenocytes [59], Murine ischaemic stroke model [93], Primary human asthmatic ASMCs [111], Primary human ASMCs [113], Human UVECs [116]
TNF-α [40,61]	↓	Human RRMS PBMCs [16,17,19,21,22], Murine splenocytes [59,115], Human HIV-infected monocyte-derived macrophages [92], Murine EAN & macrophage cell line [94], Murine hepatotoxicity model [101], Murine BMDMs [117], Murine hepatic injury & Kupffer cells [119], Murine BMDCs & allogeneic splenic T cell co-culture [122], Healthy human PBMCs [123], Murine colitis model [126], Murine epilepsy model [127], Primary murine microglial & astroglial co-cultures [128,129], Murine & primate SCD models [135], Murine renal/liver I/R injury model [136,137], Murine experimental sepsis [139], Primary murine microglia [142]
TNF-β [61,62]	↓	Murine BMDMs [117]
VEGF [40]	↓	Murine ischaemic stroke model [93], Murine osteoblastic cells [106], Murine splenocytes [115], Murine liver I/R injury model [137]

Abbreviations: ASMC: Airway smooth muscle cell; BMDC: Bone marrow-derived cells; BMDM: Bone marrow-derived macrophages; CBDMC: Cord blood-derived mast cells; DMF: Dimethyl fumarate; EAE: Experimental autoimmune encephalomyelitis; EAN: Experimental autoimmune neuritis; G-CSF: Granulocyte-colony stimulating factor; GM-CSF: Granulocyte-macrophage-colony stimulating factor; IDD: Intervertebral disc degeneration murine; IFN: Interferon; IL: Interleukin; I/R: Ischemia/reperfusion; MCP: Monocyte chemoattractant protein; MIG: Monokine induced by gamma interferon; MIP: Macrophage inflammatory protein; PBMC: Peripheral blood mononuclear cells; PDGF-BB: Platelet derived growth factor-BB; RANTES: Regulated on Activation, Normal T Cell Expressed and Secreted; RRMS: Relapsing-Remitting Multiple Sclerosis; SCD: Sickle cell disease; TNF: Tumour necrosis factor; UVEC: Umbilical vascular endothelial cells; VEGF: Vascular endothelial growth factor. Note: DMF increases IL-4 and IL-10 which are anti-inflammatory cytokines- these cytokines are already elevated in COVID-19.
